# Construction of brain atlases based on a multi-center MRI dataset of 2020 Chinese adults

**DOI:** 10.1038/srep18216

**Published:** 2015-12-18

**Authors:** Peipeng Liang, Lin Shi, Nan Chen, Yishan Luo, Xing Wang, Kai Liu, Vincent CT Mok, Winnie CW Chu, Defeng Wang, Kuncheng Li

**Affiliations:** 1 Department of Radiology, Xuanwu Hospital, Capital Medical University, Beijing 100053, China; 2 Beijing Key Laboratory of Magnetic Resonance Imaging and Brain Informatics, Beijing 100053, China; 3 Key Laboratory for Neurodegenerative Diseases, Ministry of Education, Beijing 100053, China; 4 Department of Medicine and Therapeutics, The Chinese University of Hong Kong, Shatin, NT, Hong Kong SAR.; 5 Research Center for Medical Image Computing, Department of Imaging and Interventional Radiology, The Chinese University of Hong Kong, Shatin, New Territories, Hong Kong, China.; 6 Department of Imaging and Interventional Radiology, The Chinese University of Hong Kong, Shatin, New Territories, Hong Kong, China.

## Abstract

Despite the known morphological differences (e.g., brain shape and size) in the brains of populations of different origins (e.g., age and race), the Chinese brain atlas is less studied. In the current study, we developed a statistical brain atlas based on a multi-center high quality magnetic resonance imaging (MRI) dataset of 2020 Chinese adults (18–76 years old). We constructed 12 Chinese brain atlas from the age 20 year to the age 75 at a 5 years interval. New Chinese brain standard space, coordinates, and brain area labels were further defined. The new Chinese brain atlas was validated in brain registration and segmentation. It was found that, as contrast to the MNI152 template, the proposed Chinese atlas showed higher accuracy in hippocampus segmentation and relatively smaller shape deformations during registration. These results indicate that a population-specific time varying brain atlas may be more appropriate for studies involving Chinese populations.

The creation and application of brain atlases are of great significance to brain and cognitive science^1^,^2^. With the brain atlas, it is possible for researchers to compare and/or combine the brain imaging findings from different imaging modalities (functional or structural, e.g., functional magnetic resonance imaging (fMRI), voxel-based morphology (VBM), and diffusion tensor imaging (DTI)), different brain states (healthy or diseased, e.g., Alzheimer’s disease (AD) and Parkinson’s disease (PD)), and different laboratories around the world^1^. The brain atlas is thus considered as the infrastructure of brain science studies and has been widely used in brain mapping studies. Specifically, given the reported morphological differences (e.g., brain shape and size) in the brains of populations of different origin^3^ (e.g., age, gender, race), Chinese brain template is in urgent need of supporting neuroscience researches and clinical applications concerning Chinese population.

Since the Talairach and Tournoux atlas, many brain templates have been constructed. The Talairach and Tournoux atlas is the most commonly used human brain template, which was developed based on postmortem sections of a 60-year-old French female, with the slice space ranging from 3 to 4 mm^4^. To address the limitations of the Talairach and Tournoux atlas, the Montreal Neurological Institute (MNI) and the International Consortium for Brain Mapping (ICBM) constructed a series of brain template based on several hundred western individuals’ data^5^, including MNI-305^6^, MNI152^5^ and ICBM452 (http://www.loni.usc.edu/atlases/Atlas_Detail.php?atlas_id=6). Of these brain atlases, MNI152 is the most widely used since it is included in some popular neuroimaging analysis packages such as the statistical parametric mapping package (SPM, http://www.fil.ion.ucl.ac.uk/spm/) and the expanded FMRIB Software Library (FSL, http://fsl.fmrib.ox.ac.uk/fsl/fslwiki/). Additionally, some researchers used multiple MRI scans of one single western subject to make the brain template, such as Colin-27^7^ and a French template^8^. Some attempts have also been made to establish the eastern brain template. A Korean brain template was set up using MRI and PET images of 78 normal Korean subjects^9^. Although these efforts made to build the standard brain atlas, there are still insufficiencies in these studies. First, as demonstrated by many previous studies^3^,^10^,^11^, the human brain is highly variable among phenotypically different groups (i.e., race) with fundamental genetic and environmental disparities in brain morphology and microstructure (e.g., shape, size and volume). Thus, the brain atlas of western population or other races cannot be used in Chinese populations due to the potential bias and error in brain localization. Second, all the previous brain atlases are static, which did not capture the brain atlas as a function of age and gender^11^.

In 2010, a Chinese brain atlas was also created from high-quality brain MRI scans of 56 Chinese male volunteers (aged from 20 years to 30 years), i.e., Chinese_56^3^. However, this brain atlas was constructed based on a limited sample size, and thus showed inadequate representativeness (as no female and elder subjects were included). Recently, we conducted a pilot study to develop a probabilistic MRI brain anatomical atlas based on 1000 Chinese healthy subjects (ranged from 18 years to 70 years)^12^. Ten Chinese brain atlases for different ages and genders were constructed using MR anatomical images based on HAMMER (Hierarchical Attribute Matching Mechanism for Elastic Registration)^13^ for each age and gender group (i.e., male/female with a age range of 18–30, 31–40, 41–50, 51–60 and 61–70). However, these atlases were still not ready for practical applications due to some deficiencies. First, for each atlas, a brain image with intact brain structures and global brain symmetry was chosen to serve as an initial template. This methodological limitation may drive the standard brain template bias to individual brain. Second, although the same type of scanners (1.5T MR scanners, Sonata Siemens Medical Systems, Erlangen, Germany) and sequence parameters were used, the data of 1000 subjects were collected from multi-sites with different operators. Thus, data standardization should be applied in the preprocessing stage to reduce the potential bias effects of multi-center.

The aim of the current study is to develop the Chinese adult brain templates based on a multi-center, large scale dataset (over 2000 subjects), which is nation-wide study and covers Han Chinese over a variety of regions to reduce the bias to specific region. Particularly, as compared to the previous Chinese brain atlases^3^,^12^,^14^ the new atlases based on the larger sample size may represent the brain characteristics of the Chinese population more adequately. The resulting Chinese brain templates are statistical templates, and are customized for different age and gender (http://www.chinese-brain-atlases.org).

## Results

### Chinese brain template and its different varieties

**Figure 1:**
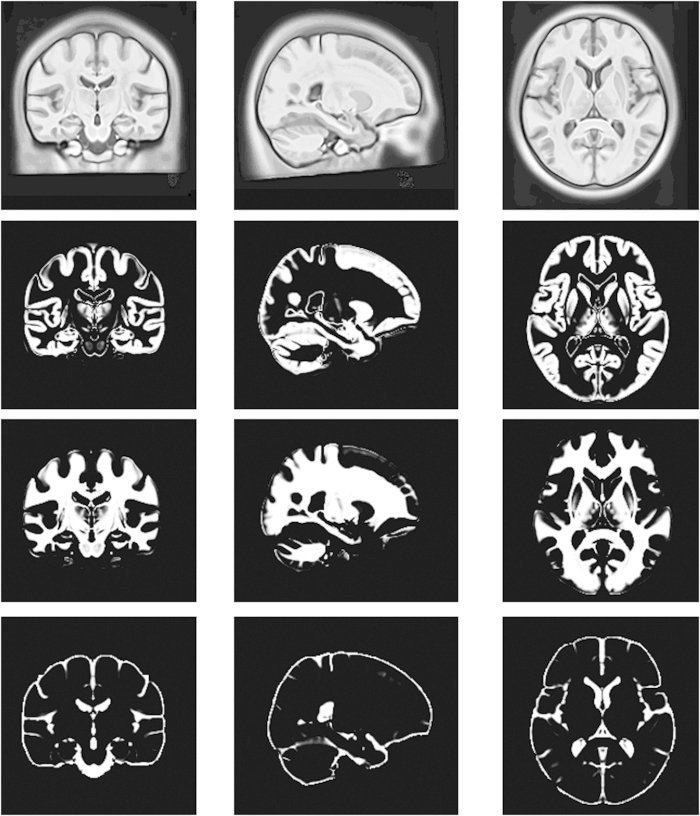
The final statistical Chinese brain template (i.e., Chinese2020) of the whole population together with its tissue probability map.

**Figure 2:**
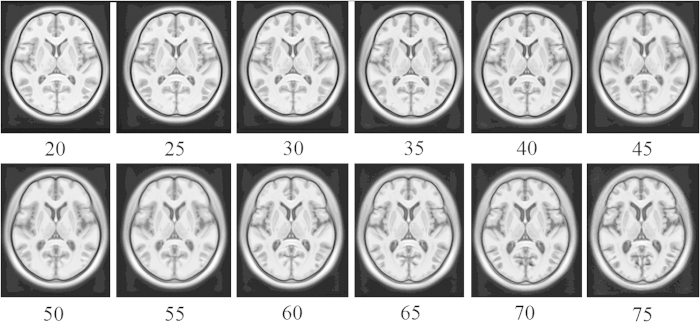
The 12 Chinese brain templates from the age 20 year to the age 75 year at a 5 years interval.

A probabilistic atlas of Chinese brain is shown in Fig. 1, named as Chinese2020, which represents the final statistical Chinese brain template (SCBT) of the whole population together with its tissue probability map. Additionally, as shown in Fig. 2, twelve probabilistic atlases of Chinese brain are presented in axial views (with the age of 20 25 30 35 40 45 50 55 60 65 70 75 years old). We further defined new Chinese standard space, coordinates, and brain area labels. These atlases have been made available online for non-commercial free download (http://www.chinese-brain-atlases.org).

### Differences between Chinese2020 and the other brain templates

**Figure 3:**
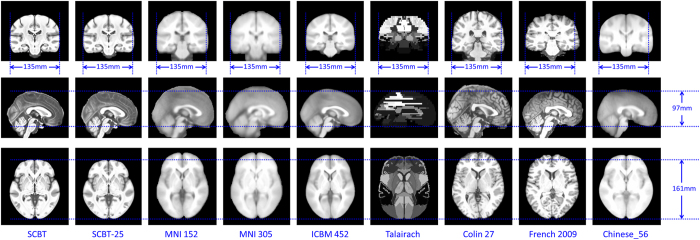
The comparison of brain shape and size between Chinese2020, SCBT-25, MNI152, ICBM305, ICBM452, Talairach, Colin27, French 2009, and Chinese_56.

The size and shape of the brain in each template as measured by the AC (anterior commissure)-PC (posterior commissure) line distance, length, width, height, and the ratios of width/length, height/length, and height/width are summarized in Table 1. Generally, compared with the templates based on Caucasian population, the SCBT template showed smaller length and height while comparable width, thus contributing to a bigger width/length ratio and a “rounder” appearance of SCBT template (Fig. 3). As contrast to the previous template based on Chinese population, i.e., Chinese_56, the SCBT was smaller in each measurement of the three directions, especially in length and width. Besides, among all the measurements, the height/length ratio and AC-PC distance are most constant across all the templates.

### Validation results of Chinese2020 in Chinese population study

#### Experiment 1

After comparing the image registration to the Chinese template (Chinese2020 and SCBT-30) and to the MNI152 template, it was found that more deformations were required in brain shape and size to register the ten new Chinese brains to the ICBM template than to the two Chinese templates (Table 2). Additionally, we also found that more significant deformations were required to register these new Chinese brains (with the mean age of 27.75) to Chinese2020 than to SCBT-30. These results reveal that the Chinese templates, especially the age-matched Chinese template (SCBT-30 in this validation experiment) better represents the shape and size of the Chinese population.

#### Experiment 2

Structural MRI of 20 Chinese subjects were automatically segmented using both our Chinese atlas and the AAL atlas. We also manually segmented these Chinese subjects to serve as the ground truth segmentation. The accuracy of atlas-based segmentation was measured in terms of Dice similarity coefficient (DSC), which measures the degree of volume overlap of the manual and automatic segmentations. DSC is defined as:


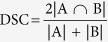


where A and B are two volumes under comparison. DSC ranges between 0 and 1, where 1 indicates perfect matching. It was found that the DSC is 0.7584 ± 0.0396 using our Chinese atlas and 0.6987 ± 0.0435 using AAL atlas. The segmentation results have been significantly improved by using our Chinese atlas (*P* = 0.001).

## Discussion

In this paper, new Chinese brain atlases were constructed and validated using a multi-center MRI dataset of 2020 Chinese adults. The spatial resolution of the Chinese templates is 1 × 1 × 1 mm^3^ and all templates are open accessable and freely downloadable (http://www.chinese-brain-atlases.org).

### The effectiveness of the template construction method

In order to make the study more rigorous and effective, we have reduced human intervention during template construction as much as possible. The large and widespread brain MRI database renders the study more representative and unbiased, nevertheless, it also brings some challenges. For instance, some images have quite low image quality. Incorporation of these low quality images would adversely affect the quality of the template. Thus, we adopted an automatic noise estimation method to first exclude those images with a quite low signal-to-noise ratio (SNR). Furthermore, in each iteration of normalization, we did not select any specific subject but use the average brain as the initial reference, so as to reduce the bias during template construction.

Our MRI data come from multiple hospitals in a nationwide scale. Although we have set a consistent standard for image acquisition, these hospitals may use different machines for MRI acquisition. There exists large variance in the intensity profiles of MRIs obtained by different machines. In order to make consistent analysis of these images, intensity profile was normalized before further processing. To handle the large database, we took an automatic histogram matching method to normalize the histogram of each subject to that of a standard template. After normalization, the histograms of different subjects are, by and large, in the same intensity range.

### The effectiveness of using Chinese2020

Given the known brain morphometric and volumetric differences between Chinese and Caucasian populations^3^,^15^, the Chinese brain template should be used in the neuroimaging studies of Chinese population due to the potential registration bias and localization deviation when using the Caucasian template as the reference template. This study has further demonstrated the differences between Chinese and Caucasian observed in the previous studies (Fig. 3). Specifically, fewer deformations were required to register the Chinese subjects’ brains to Chinese2020 and SCBT-30 than to MNI152 using a 12-parameter transformation, which suggests that the Chinese brain template better represents the brain characteristics of the Chinese population. Moreover, it was also verified that, in contrast to use the Caucasian template, hippocampus segmentation of Chinese subjects showed significant higher accuracy when using the Chinese2020 template. Additionally, the current Chinese brain atlas, i.e., Chinese2020, might have the better generalization ability to multi-sites and multi-scanner than the other Chinese templates, as it was built on a multi-center, large scale Chinese population.

### The advantage of the dynamic template

Different from the static brain atlas previously reported (e.g., Chinese_56), we presented a series of brain templates for 12 different age bracket (20, 25, 30, 35, 40, 45, 50, 55, 60, 65, 70 and 75) as shown in Fig. 2. Actually, by using the identical template construction method, we could customize the Chinese brain template for every age between 18 and 76. This kind of customized brain template, but not a common template as implemented in SPM and AFNI now, is preferable for a specific study, as age has a significant effect on brain structure and morphological characteristics^16^. This has been further demonstrated in this study, as SCBT-30 better represents the group of young Chinese subjects than Chinese2020 (Table 2). In the future, users could submit their requirement of the Chinese brain template with the specific age and gender (i.e., for a specific study) online (http://www.chinese-brain-atlases.org), and the new brain template would be computed and returned.

It was argued that group-specific brain template for a specific study could be built based on structural MRI of all subjects. However, due to the relatively limited sample size, this kind of group-specific template might have lower SNR and statistical power, thus have worse representativeness. In addition, these group-specific templates may induce new bias between different studies. In contrast, our dynamic brain templates for different ages were built based on a large scale multi-center datasets, which better represent the brain characteristics of the Chinese population of different ages than the static brain template (e.g., Chinese_56).

### Future directions

The current Chinese brain template (Chinese2020) is constructed using 1.5 MRI scans with the spatial resolution of 1 × 1 × 1 mm^3^. The next updating of the Chinese brain template may use 3.0T MRI acquisition protocols in order to capture more detailed and precise anatomical information in the brain template. As is known, except age, gender, ethnicity and disease condition (e.g., AD and PD) may also exert on brain structures and functions. Thus, new Chinese brain templates especially applicable to the corresponding sub-populations in terms of age, gender, ethnicity, and disease condition should be built in the future.

## Materials and Methods

### Subjects

Two thousand nine hundred healthy adults from 24 provinces of China were recruited by 15 hospitals. Medical examinations were conducted to exclude subjects with a lifetime history of any neurological, psychiatric, or significant medical illnesses as well as patients with a past history of substance abuse. All participants gave their written informed consents before the experiment were performed. Seven hundred and forty participants were excluded due to missing information or invalid brain imaging data. One hundred and forty subjects’ data were further excluded due to the high noise level in the images. Finally, 1081 valid participants’ data (559 females, mean age = 44.3 years, range =18–76) were used among those scanned in Siemens scanner and 939 valid participants’ data (515 females, mean age = 42.4 years, range = 18–74) were analyzed among those scanned in GE scanner. This study was approved by the Ethics Committee of Xuanwu Hospital, Capital Medical University. The methods were carried out in accordance with the approved guidelines.

### Image acquisition

All 15 hospitals participating in this study followed the same recruitment procedure and the same MR protocols (either Siemens system or GE system). Three-dimensional high-resolution T1-weighted anatomical images were acquired by using an 8-channel phased array head coil. Scanning was performed on a 1.5 Tesla MRI system (Siemens Medical System, Erlanger, Germany) by using a T1 weighted 3D MPRAGE sequence (TR/TE = 2000/4ms, matrix = 512 × 512, 15° flip angle, slice thickness = 1mm, 192 sagittal slices), or a GE Signa HDx 1.5T scanner (Fairfield, US) by using a Spoiled Gradient Recalled Echo (SPGR) sequence (TR/TE = 2000/4 ms, matrix = 256 × 256, 15° flip angle, slice thickness = 1 mm, 146 sagittal slices). Foam padding and headphones were used to limit head motion and reduce scanning noise. The quality of each brain volume has been ensured to be in good condition and without observable brain abnormality by an experienced radiologist.

### Data Pre-processing

To preprocess T1 MRIs, apart from traditional schemes, such as bias field correction using N4ITK and brain orientation adjustment, we have also designed an intensity normalization method to address the intensity profile difference due to different acquisition machines used by different hospitals, and an automatic noise estimation method to control the quality of incorporated images. As brain MRIs in our project were collected from different hospitals and using different scanners (either GE or Siemens), there are distinctive intensity range difference between these images. Matching the intensity profiles of different images from different acquisitions can be good for improving image registration accuracy. Therefore, we have adopted a histogram matching scheme to normalize the histogram of each subject to a standard histogram of a template image, where the histogram of Colin27 was used as the standard histogram in our study due to its high resolution and high signal to noise ratio. Furthermore, although the image acquisition procedure followed strict standard, there are still some images have quite low image quality. The inclusion of these noisy images in template construction may adversely impact the quality of the templates. Therefore, we applied an automatic noise estimation method^18^ for image quality assessment. A threshold of noise level is set to screen out those unacceptable noisy images.

### Template construction

We have constructed 12 templates from the age 20 year to the age 75 year at a 5 years interval. To generate a template from the group of images, inter-subject linear registration was performed to bring the images into the common space. We used the SyN algorithm in ANTS software^19^ to implement image registration. During registration, to exclude the effects of background noise in matching efficiency, we employed a mask scheme in registration, where only voxels in brain mask have been accounted for similarity metric calculation. After registration, a temporary template was built using the normalized images. A kernel regression scheme was taken to build the template. That is, the subject with a closer age with respect to the template age will contribute more than those subjects away from the template in age. This kernel regression scheme can help solve the problem of missing subject at a certain age or uneven age distribution. After producing the 12 brain templates for age group from 20 to 75 years old, we also created a whole population brain template serving as the Chinese brain standard space. Therefore, in the template level, we continue to perform non-rigid registration to bring the 12 templates into one common space and create the final brain template.

### Validation of the new atlas in Chinese population study

Two experiments were run to validate the use of the new Chinese atlas (i.e., Chinese2020). In the following validation context, except the selection of the template for registration and segmentation (Chinese2020, SCBT-30, MNI152, and AAL atlas), all the other protocols were kept the same.

#### Experiment 1

Brain MRI volumes of ten new Chinese subjects (5 male, 27.75 ±2.84) were aligned to the Chinese brain template (Chinese2020, SCBT-30) and the MNI152 atlas respectively using a 12-parameter transformation as implemented in SPM5. The brain global features was then statistically compared between the original brain (i.e., in the native space) and the brains registering to Chinese2020, between the original brain and the brains registering to SCBT-30, as well as between the original brain and the brains registering to MNI152, by using a paired t test. Thus, the deformations during the image registering to the two Chinese templates and to the MNI152 template could be quantitatively evaluated.

#### Experiment 2

Atlas-based segmentation of hippocampus is a widely used method for automatic segmentation of hippocampus^20^,^21^. The hippocampus of an MRI template was first manually labeled by an expert rater. The MRI template was then matched to the subject MR image using non-rigid registration method. The resulting non-rigid transformation was applied to propagate the manual hippocampus labels in the template image to the target subject image space, serving as the automatic segmentation result for this subject. This procedure was called atlas-based segmentation. In current studies, one of the most widely used atlas for hippocampus segmentation is the AAL atlas^22^, which has been embedded in the SPM toolbox. However, AAL atlas was constructed based on Caucasian brain data. The morphometric differences between Chinese and Caucasian may induce the inaccuracy of segmenting hippocampus of the Chinese population using the Caucasian atlas.

We have conducted experiments to validate the superiority of using our constructed Chinese atlas for hippocampus segmentation of Chinese population. We compared the hippocampus segmentation results using our Chinese atlas with the results using AAL atlas. We used SyN in ANTs (http://www.picsl.upenn.edu/ANTS/) for registration. The registration parameters were set as the same for all the subjects and atlases. Cross correlation was used as similarity metric. A Gaussian regularizer with a sigma of 3 was operated on the deformation field. The optimization will be performed over three resolutions, with a maximum of 50 iterations at the first two coarse levels and 10 iterations at the full resolution level.

## Additional Information

**How to cite this article**: Liang, P. *et al.* Construction of brain atlases based on a multi-center MRI dataset of 2020 Chinese adults. *Sci. Rep.* **5**, 18216; doi: 10.1038/srep18216 (2015).

## Figures and Tables

**Table 1:** The comparisons of brain shape and size between Chinese2020 and the other atlases.

Measurement (mm)	Chinese2020	SCBT-25	MNI 152	MNI 305	ICBM 452	Talairach	Colin27	French (2009)	Chinese_56
AC-PC	26	26	28	28	28	—	27	27	26
Length	161	159	177	176	172	170	177	173	171
Width	135	138	138	135	130	137	141	144	144
Height	97	97	109	108	103	98	113	100	101
Width/Length	0.84	0.87	0.78	0.77	0.76	0.81	0.80	0.83	0.84
Height/Length	0.60	0.61	0.62	0.61	0.60	0.58	0.64	0.58	0.59
Height/Width	0.72	0.70	0.79	0.80	0.79	0.72	0.80	0.69	0.70

Note: SCBT-25 represents the Chinese atlas of 25 years old group.

**Table 2:** Brain shape and size differences registering separately the 10 additional (5 male) Chinese subjects into the MNI152 and the Chinese2020 atlas spaces.

Measurement (mm)	Original brains	Registered to MNI152	Registered to Chinese2020	Registered to SCBT-30	*P*values		
					*P1*	*P2*	*P3*
AC-PC	25.51 ± 1.12	29.20 ± 0.98	26.00 ± 1.26	25.41 ± 1.28	<0.001*	0.443	0.877
Length	149.60 ± 7.02	173.60 ± 5.04	156.00 ± 3.58	154.60 ± 2.54	<0.001*	0.011	0.076
Height	114.20 ± 4.47	121.60 ± 2.33	114.40 ± 5.04	115.80 ± 2.27	0.003*	0.913	0.408
Width	137.30 ± 6.31	137.60 ± 2.80	133.40 ± 2.01	134.60 ± 2.20	0.914	0.107	0.319
Width/Length	0.92 ± 0.06	0.79 ± 0.03	0.86 ± 0.02	0.87 ± 0.02	<0.001*	0.003*	0.020
Height/Length	0.77 ± 0.05	0.70 ± 0.03	0.73 ± 0.04	0.75 ± 0.02	0.002*	0.075	0.376
Height/Width	0.83 ± 0.04	0.88 ± 0.02	0.86 ± 0.04	0.86 ± 0.03	0.016	0.250	0.159

Note: *P1* was the statistical significance for the measured values of original brains and the brains registering to the MNI152 atlas. *P2* was the statistical significance for the measured values of original brains and the brains registering to the Chinese2020 atlas. *P3* was the statistical significance for the measured values of original brains and the brains registering to the SCBT-30 atlas. *P < 0.01.
